# Human engineered cardiac tissue model of hypertrophic cardiomyopathy recapitulates key hallmarks of the disease and the effect of chronic mavacamten treatment

**DOI:** 10.3389/fbioe.2023.1227184

**Published:** 2023-09-08

**Authors:** Kai Wang, Brian J. Schriver, Roozbeh Aschar-Sobbi, Alex Y. Yi, Nicole T. Feric, Michael P. Graziano

**Affiliations:** Valo Health, Inc., Department of Discovery Research, New York, NY, United States

**Keywords:** hypertrophic cardiomyopathy, human induced pluripotent stem cells derived cardiomyocyte, engineered cardiac tissue, disease model, mavacamten

## Abstract

**Introduction:** The development of patient-specific induced pluripotent stem cell-derived cardiomyocytes (iPSC-CMs) offers an opportunity to study genotype-phenotype correlation of hypertrophic cardiomyopathy (HCM), one of the most common inherited cardiac diseases. However, immaturity of the iPSC-CMs and the lack of a multicellular composition pose concerns over its faithfulness in disease modeling and its utility in developing mechanism-specific treatment.

**Methods:** The Biowire platform was used to generate 3D engineered cardiac tissues (ECTs) using HCM patient-derived iPSC-CMs carrying a β-myosin mutation (MYH7-R403Q) and its isogenic control (WT), withal ECTs contained healthy human cardiac fibroblasts. ECTs were subjected to electro-mechanical maturation for 6 weeks before being used in HCM phenotype studies.

**Results:** Both WT and R403Q ECTs exhibited mature cardiac phenotypes, including a lack of automaticity and a ventricular-like action potential (AP) with a resting membrane potential < −75 mV. Compared to WT, R403Q ECTs demonstrated many HCM-associated pathological changes including increased tissue size and cell volume, shortened sarcomere length and disorganized sarcomere structure. In functional assays, R403Q ECTs showed increased twitch amplitude, slower contractile kinetics, a less pronounced force-frequency relationship, a smaller post-rest potentiation, prolonged AP durations, and slower Ca^2+^ transient decay time. Finally, we observed downregulation of calcium handling genes and upregulation of *NPPB* in R403Q vs. WT ECTs. In an HCM phenotype prevention experiment, ECTs were treated for 5-weeks with 250 nM mavacamten or a vehicle control. We found that chronic mavacamten treatment of R403Q ECTs: (i) shortened relaxation time, (ii) reduced APD_90_ prolongation, (iii) upregulated *ADRB2*, *ATP2A2*, *RYR2,* and *CACNA1C*, (iv) decreased B-type natriuretic peptide (BNP) mRNA and protein expression levels, and (v) increased sarcomere length and reduced sarcomere disarray.

**Discussion:** Taken together, we demonstrated R403Q ECTs generated in the Biowire platform recapitulated many cardiac hypertrophy phenotypes and that chronic mavacamten treatment prevented much of the pathology. This demonstrates that the Biowire ECTs are well-suited to phenotypic-based drug discovery in a human-relevant disease model.

## 1 Introduction

Hypertrophic cardiomyopathy (HCM) is one of the most common inherited cardiac diseases with an estimated worldwide prevalence of 1:200–1:500 (Semsarian et al., 2015; Maron et al., 2022a). Genetic studies have identified over 1500 HCM-associated mutations, mostly missense, spreading across more than 20 genes that encode cardiac sarcomeric or Z-disc proteins. About 80% of these mutations are found in either the *MYH7* or *MYBPC3* gene, the corresponding proteins of which are responsible for the generation or regulation of cardiac contractility (Alfares et al., 2015). Much like HCM genetics, the disease severity is highly variable, ranging from asymptomatic to early-onset heart failure or sudden cardiac death at a young age. HCM is primarily diagnosed by the thickening of the ventricular septum and left ventricular (LV) free wall by 15 mm or more in an echocardiogram or other imaging approaches (Maron et al., 2022a). Elevated LV systolic contractile function and impaired diastolic function are also key physiological features of HCM. Histological studies often find hypertrophied myocytes, regions of myocyte disarray and interstitial fibrosis within the thickened myocardium (Marian and Braunwald, 2017). The scale of these histological changes generally reflects the severity of the disease and are associated with clinical course and management (Varnava et al., 2001; Galati et al., 2016; Cui et al., 2021).

The R403Q mutation in *MYH7*, the gene encoding for β-myosin heavy chain, was the first identified HCM mutation and is associated with severe clinical phenotypes including early-onset, progressive myocardial dysfunction, and a high incidence of cardiac sudden death (Geisterfer-Lowrance et al., 1990; Lopes et al., 2013). While some of these phenotypes were observed in animal models carrying the R403Q mutation (Geisterfer-Lowrance et al., 1996; Nagueh et al., 2004; Anderson et al., 2018), phenotypic inconsistencies between models were also reported (Tyska et al., 2000; Lowey et al., 2018). This lack of consistency has made characterizing the pathogenic mechanism or predicting a therapeutic outcome in humans very challenging (Vakrou et al., 2021).

The recent development of robust cardiac differentiation protocols for human induced pluripotent stem cells (hiPSCs) created the potential for an unlimited supply of human derived cardiomyocytes (CMs) from both healthy and diseased sources, which in turn has enabled investigations into species-to-species differences and provided an alternative to costly animal studies (Protze et al., 2019; Rowe and Daley, 2019). However, major concerns over using hiPSC-CMs for disease modeling have also been reported. Most studies of this type use 2D cultures of hiPSC-CMs shortly after cardiac differentiation (Mosqueira et al., 2019; Vučković et al., 2022), generating concerns over the immaturity of the cells relative to adult CMs, and the lack of a multicellular composition. To address these issues, many researchers started using 3D tissue models composed of different cell types (Liu et al., 2018), ourselves included. We were able to demonstrate that 3D engineered cardiac tissues (ECTs) generated on the Biowire platform and composed of healthy hiPSC-CMs and cardiac fibroblasts acquired adult-like contractile properties and responses to inotropic agents after long-term electrical stimulation (Feric et al., 2019; Zhao et al., 2019).

In the present study, ECTs were generated from patient-derived iPSC-CMs carrying the MYH7-R403Q mutation and its genetically corrected isogenic control (WT). The pathogenic role of the mutation was systematically assessed in the ECTs in terms of morphology, contractility, Ca^2+^ handling, electrophysiology and gene expression. Compared to WT, R403Q ECTs exhibited pathological and physiological changes that faithfully reproduced key disease features observed for HCM patients (Burchfield et al., 2013). Additionally, we found that treating ECTs for 5 weeks with mavacamten, an FDA-approved treatment for HCM, partially reversed the pathological phenotypes of the R403Q mutation, including an improvement in the relaxation properties. Importantly, our findings are consistent with the therapeutic effects of mavacamten reported for animal models and in clinical trials (Green et al., 2016; Olivotto et al., 2020).

## 2 Materials and methods

### 2.1 Generation and maturation of ECTs

The hiPSC-CMs carrying the HCM-related mutation MYH7-R403Q (R403Q) and the isogenic control hiPSC-CMs (WT) were both purchased from FUJIFILM Cellular Dynamics (Madison, WI, USA). R403Q hiPSC-CMs were derived from an HCM patient carrying the MYH7-R403Q mutation and were previously reported as a tool for HCM disease modeling (Dainis et al., 2020). The MYH7-R403Q mutation in the patient’s iPSCs was corrected via gene-editing to generate the isogenic control line. Human ECTs were generated in the Biowire platform, an 8-well polystyrene strip containing parallel poly (octamethylene maleate (anhydride) citrate) (POMaC) wires, as previously reported (Feric et al., 2019; Zhao et al., 2019). Briefly, hiPSC-CMs were thawed and mixed with freshly isolated human ventricular cardiac fibroblasts (hvCBs; Lonza, NJ, USA) in a 10:1 ratio, pelleted and re-suspended at a concentration of 5.5 × 10^7^ cells/mL in a collagen/Matrigel/fibrin hydrogel formed by mixing 75% v/v collagen hydrogel (rat tail collagen, 3 mg/mL, Corning, AZ, USA; 1x M199; Matrigel, 15% v/v, Corning; deionized H_2_O; NaOH, 0.01N; NaHCO_3_, 0.22 mg/mL) and 25% fibrinogen hydrogel (fibrinogen, 33 mg/mL; HEPES, 20mM; NaCl, 0.9% w/v). The cell suspension (2 µL) was seeded into the Biowire platform resulting in 1.1 × 10^5^ cells per ECT, an optimized number with greater than 90% ECT yield. The seeded cells were cultured in plating media (FUJIFILM Cellular Dynamics, WI, USA) containing 0.01 mg/mL aprotinin at 37°C and 5% CO_2_ for 7 days to form a compacted ECT suspended between 2 POMaC wires. To enable electrical field stimulation, each polystyrene strip with ECTs was placed in a 10 cm electrode dish (Zhao et al., 2019), and switched to Induction 3 Medium (StemPro-34 complete media, ThermoFisher Scientific, MA, USA; 20 mM HEPES; 1% GlutaMAX, ThermoFisher Scientific; 1% Penicillin-Streptomycin, ThermoFisher Scientific; 213 μg/mL 2-phosphate Ascorbic Acid). Stimulation frequency started at 1 Hz and increased daily to a maximum of 5.2 Hz, while the voltage was set at 2x the average excitation threshold of the ECTs. At the end of week 7, the electrical stimulation frequency was reduced to 3 Hz for the remainder of the ECT culture. Contractility, electrophysiology, Ca^2+^ imaging and acute pharmacology experiments were conducted on ECTs that had been electrically stimulated at 3 Hz for a minimum of 7 days. All chemicals were purchased from Sigma-Aldrich (MO, USA) unless otherwise specified.

### 2.2 ECT area measurements from bright field images

ECT areas were computed from brightfield images acquired at 2x. For each image, the ECT contour was automatically segmented using a purpose-built software (Python). Briefly, greyscale images were automatically pre-processed, which included having their contrasts adjusted, asymmetrically Gaussian smoothed to blur high contrast POMaC wires, and normalized, followed by binarization using a dynamic threshold. ECT segmentation was performed on the binary images by combining a series of denoising, edge-based, and morphological operations. For every image, the contour was overlaid and visually inspected to ensure it accurately bordered the ECT. Poor segmentations were not included in further analysis. The total number of pixels within the segmented ECT contour was computed and then converted from total number of pixels to area in mm^2^.

### 2.3 Immunostaining and confocal imaging of ECTs and sarcomere measurements

ECTs were first incubated in Ca^2+^ free Tyrode’s solution supplemented with 2 mM BAPTA for 5 min and then fixed in 4% paraformaldehyde (PFA) for 20 min. After permeabilization and blocking in PBS with 0.3% Triton X-100, 1% BSA and 1% donkey serum, ECTs were stained with mouse anti-cardiac Troponin-T (2 μg/mL; MS295P1, Epredia, MI, USA) and rabbit anti-α-actinin2 (1.4 μg/mL; EP2529Y, Abcam, MA, USA) primary antibodies at 4°C overnight followed by staining with donkey anti-mouse-Alexa Fluor 488 (1:1,000) and donkey anti-rabbit-Alexa Fluor 594 (1:1,000) secondary antibodies at room temperature for 2 h. DAPI was used for staining the nuclei. Z-stack images (10–20 µm from the surface of the ECT) of ECTs were acquired using a spinning disk confocal microscope (Nikon, NY, USA) with a ×60 oil objective at a 0.2 µm step-size. To measure sarcomere parameters, Z-disc segmentation was carried out based on the α-actinin2 signal following a previously published protocol (Supplementary Figure S1) (Zhao et al., 2021). Briefly, the contours of the Z-disc were first found by computing the Laplacian of Gaussian of an image using scikit-image (Python) (Supplementary Figures S1Ai, Bi). Subsequently, sarcomeres were identified by linking approximately parallel Z-discs (Supplementary Figures S1Aii, Bii). For each sarcomere within a single image, sarcomere length was computed as the distance between the two associated Z-disc centers. The median value for each image was computed, and the mean of all images within a group was then reported. Sarcomere angle was computed as the angle of the vector connecting the two associated Z-discs. The standard deviation was computed for each image, and the mean of all images within a group was then reported.

### 2.4 Isolation of cardiomyocytes from ECTs and individual cell volume measurements

ECTs were removed from the Biowire platform and enzymatically digested by incubating with collagenase II (200 units/mL, ThermoFisher, MA, USA) and protease XIV (0.1 units/mL, Sigma-Aldrich, MA, USA) for 30–40 min at 37°C in a modified Tyrode’s buffer containing (in mmol/L): 120 NaCl, 5.4 KCl, 5 MgSO_4_, 5 sodium pyruvate, 20 glucose, 20 taurine, 10 HEPES, 30 butanedione monoxime, pH 6.9. Isolated cardiomyocytes (CMs) were pelleted at 200 x g for 3 min and resuspended in Smear Gell (Diagnocine, NJ, USA). Smear Gell with isolated CMs was spread on glass slides at about 100 cells/cm^2^ and solidified according to the manufacturer’s protocol. CMs were fixed on the glass slides in 4% PFA for 20 min. After permeabilization and blocking in PBS with 0.3% Triton X-100, 1% BSA and 1% donkey serum, CMs were stained with mouse anti-cardiac Troponin-T (2 μg/mL; MS295P1, Epredia, MI, USA) primary antibody at 4°C overnight followed by staining with donkey anti-mouse-Alexa Fluro 488 (1:1,000) secondary antibody and Phalloidin Alexa Fluor 647 (1:500) at room temperature for 2 h. DAPI was used for staining nuclei. Z-stack images of individual CMs were acquired using an LMS880 confocal microscope (Zeiss, CA, USA) with a ×63 oil objective, a 0.5 µm step-size, and 50% interval overlap. Z-stack images of ECTs were analyzed using ImageJ software (Supplementary Video S1) (NIH, MD, USA). Cell volume of isolated CMs was measured based on cardiac Troponin-T staining using Imaris Cell software (Oxford Instruments, OX, UK).

### 2.5 Image-based contractility measurements

ECT contractility was measured by tracking the deflection of the POMaC wires as a function of time using an Eclipse Ti2 microscope with an environmental chamber (37°C, 5% CO_2_) and NIS-Elements Advanced Research software (Nikon, NY, USA) as previously described (Feric et al., 2019; Zhao et al., 2019). Using custom software, deflection of the POMaC wires in pixels and frame number were converted to force and time, respectively. This was accomplished by fitting a curve along the centerline of the fluorescent POMaC wire and a line between the anchor points at either end of the wire to indicate a reference baseline or at rest position. For each frame in the video, the distance between the curve and the reference line was computed and then converted to force based on an experimentally determined equation describing the relationship between displacement and force (Zhao et al., 2019). Each contraction in the force time-series was then fitted with a double-sigmoid model to remove noise. Contractile parameters were computed from the fitted functions.

### 2.6 Sharp-electrode action potential recordings

A single ECT was placed into an experimental chamber filled with modified Krebs Henseleit solution (in mmol/L): 118 KCl, 4.7 NaCl, 1.2 MgSO_4_, 1.2 KH_2_PO_4_, 0.2 sodium pyruvate, 11 D-glucose, 1.8 CaCl_2_, 22 NaHCO_3_ bubbled with 95% O_2_/5% CO_2_. The temperature of the solution was maintained at 37°C with a flow rate at 2.5 mL/min. The ECT was allowed to equilibrate for 30 min under point stimulation at 1.0 Hz. High impedance borosilicate microelectrodes were prepared with a tip resistance of 40–80 MΩ once filled with 3 M KCl. Upon ECT impalement, the membrane potential was allowed to stabilize for 60 s. Bipolar stimulation at 1.5x excitation threshold was applied to one end of the ECT using platinum electrodes. Signals were amplified using a Duo 773 amplifier (World Precision Instruments, FL, USA). Recordings were performed in continuous mode with sampling at 10 kHz using a Digidata 1322a digitizer and AxoScope software (Molecular Devices, CA, USA).

### 2.7 Calcium transients imaging

Calcium transients were measured from ECTs as previously described (Zhao et al., 2019), with some modification (Supplementary Videos S2, S3). Briefly, all experiments were performed in an environmental chamber at 37°C and 5% CO_2_, and recordings were obtained using an Eclipse Ti2 microscope using NIS-Elements Advanced Research software (Nikon). ECTs were incubated in serum-free maintenance media containing 20 µM Calbryte630 AM (AAT BioQuest, CA, USA) for 60 min before removing them from the dye loading buffer and transferring them into a custom Ca^2+^ imaging plate with a glass bottom. ECTs were field stimulated at 1 Hz in fresh maintenance media for 60 min before imaging. Calcium transients were measured as the average change in fluorescent intensity using custom software that calculated the amplitude and kinetics of the calcium transients. Specifically, a region of interest (ROI) was specified for the video that excluded the background and any extremely bright spots caused by possible artifacts. For each frame, the average intensity was computed for the ROI and the fluorescent time-series was then fitted with a biexponential model to remove noise. Calcium parameters were computed from the fitted functions.

### 2.8 RNA isolation and RT-qPCR

Intact ECTs were removed from the Biowire platform and individually lysed in TRIzol Reagent (ThermoFisher, MA, USA). RNA from individual ECTs was isolated using Direct-zol RNA Microprep Kit with in-column DNA digestion (Zymo Research, CA, USA) and measured using a NanoDrop One spectrophotometer (ThermoFisher, MA, USA). The quality of isolated RNA was evaluated using a high sensitivity chip and a 2,100 Bioanalyzer (Agilent, CA, USA). RNA samples with RIN > 8 were considered acceptable for transcriptome analysis. Complementary DNA was synthesized from the RNA of individual ECTs using SuperScript IV VILO master mix (ThermoFisher, MA, USA). Real-time qPCR using TaqMan probes (Table 1) and TaqMan Fast Advanced Master Mix (ThermoFisher, MA, USA) were prepared using a MicroLab Prep automated liquid handler (Hamilton, NV, USA) and performed on a QuantStudio 3 real-time PCR system (ThermoFisher, MA, USA) with duplicate reactions per probe per sample. Relative gene expression was determined using the QuantStudio Relative Qualification Analysis Module (ThermoFisher, MA, USA) and normalized to the housekeeping genes *GAPDH* and *RPL13A*.

**TABLE 1:** TaqMan assays used in RT-qPCR experiments.

Gene/alias	Description	Function	TaqMan assay ID
MYH6	Myosin Heavy Chain 6	Sarcomere protein	Hs01101425_m1
MYH7	Myosin Heavy Chain 7	Sarcomere protein	Hs01110632_m1
MYL2	Myosin Light Chain 2	Sarcomere protein	Hs00166405_m1
ADRB1	Adrenoceptor Beta 1	Adrenergic receptor	Hs02330048_s1
ADRB2	Adrenoceptor Beta 2	Adrenergic receptor	Hs00240532_s1
PLCG2	Phospholipase C Gamma 2	Phospholipases	Hs01101857_m1
CALM1	Calmodulin 1	Phosphorylase kinase	Hs00237233_m1
CAMK2D	Calcium/Calmodulin Dependent Protein Kinase II Delta	Calcium/Calmodulin-dependent protein kinase	Hs00943554_m1
PLN	Phospholamban	Protein kinase substrate	Hs01848144_s1
ATP2A2	ATPase Sarcoplasmic/Endoplasmic Reticulum Calcium Transporting 2	Calcium-transporting ATPase of sarcoplasmic reticulum	Hs00544877_m1
CASQ1	Calsequestrin 1	Calcium binding protein in sarcoplasmic reticulum	Hs00154281_m1
CASQ2	Calsequestrin 2	Calcium binding protein in sarcoplasmic reticulum	Hs00154286_m1
RYR2	Ryanodine receptor 2	Cardiac muscle Ryanodine receptor-calcium release channel	Hs00181461_m1
GATA4	GATA Binding Protein 4	Transcription Factor	Hs00171403_m1
NFATC4/NFAT3	Nuclear Factor of Activated T Cells 4	Nuclear factor	Hs00190037_m1
NPPA/ANP	Natriuretic Peptide A	Cardiac circulating marker	Hs00383230_g1
NPPB/BNP	Natriuretic Peptide B	Cardiac circulating marker	Hs00173590_m1
CACNA1C	Calcium Voltage-Gated Channel Subunit Alpha1 C	Calcium channel	Hs00167681_m1
CACNA1G	Calcium Voltage-Gated Channel Subunit Alpha1 G	Calcium channel	Hs00367969_m1
CACNA1H	Calcium Voltage-Gated Channel Subunit Alpha1 H	Calcium channel	Hs01103527_m1
HCN1	Hyperpolarization Activated Cyclic Nucleotide Gated Potassium Channel 1	Potassium channel	Hs01085412_m1
HCN2	Hyperpolarization Activated Cyclic Nucleotide Gated Potassium Channel 2	Potassium channel	Hs00606903_m1
HCN4	Hyperpolarization Activated Cyclic Nucleotide Gated Potassium Channel 4	Potassium channel	Hs00975492_m1
KCND3/Kv4.3	Potassium Voltage-Gated Channel Subfamily D Member 3	Potassium channel	Hs00542597_m1
KCNH2/HERG	Potassium Voltage-Gated Channel Subfamily H Member 2	Potassium channel	Hs04234270_g1
KCNJ2/Kir2.1	Potassium Inwardly Rectifying Channel Subfamily J Member 2	Potassium channel	Hs00265315_m1
KCNJ12/Kir2.2	Potassium Inwardly Rectifying Channel Subfamily J Member 12	Potassium channel	Hs05015288_s1
KCNQ1	Potassium Voltage-Gated Channel Subfamily Q Member 1	Potassium channel	Hs00923522_m1
KCNE1/MinK	Potassium Voltage-Gated Channel Subfamily E Regulatory Subunit 1	Potassium channel accessory subunit	Hs00264799_s1
KCNE2/MiRP1	Potassium Voltage-Gated Channel Subfamily E Regulatory Subunit 2	Potassium channel accessory subunit	Hs00270822_s1
SCN5A/Nav1.5	Sodium Voltage-Gated Channel Alpha Subunit 5	Sodium channel	Hs00165693_m1
SLC8A1/NCX1	Solute Carrier Family 8 Member A1	Sodium/Calcium exchanger	Hs01062258_m1

### 2.9 NT-proBNP measurements from conditioned media

The NT-proBNP ELISA Assay (Biomedica, Vienna, Austria) was performed according to the manufacturer’s instructions with some modifications. Briefly, conditioned media was removed from the ECTs after 4 days in culture and stored at −20°C. NT-proBNP concentrations in the conditioned media were measured on a Varioskan LUX multimode microplate reader (ThermoFisher, MA, USA) using the manufacturer’s protocol.

### 2.10 Acute treatment of ECTs

ECTs without spontaneous activity at the end of the maturation process were used for compound testing. Tests were conducted in a 37°C, 5% CO_2_ environmental chamber under field stimulation at 1 Hz. ECTs were incubated in 3 mL culture media for 30 min in the environmental chamber after which a baseline contractility video was acquired (30 s video). The test article stock solutions were prepared in either dimethyl sulfoxide or water, as appropriate, and serially diluted in medium. The first (lowest concentration) test article stock solution was then added to the well and mixed to provide the desired final concentration. Following 15 min of incubation, a 30s video was acquired. The same procedure was followed for all subsequent concentrations (lowest to highest) such that 1 ECT was incubated with all test concentrations.

### 2.11 Chronic treatment of ECTs

A mavacamten stock solution was prepared in DMSO and added to the ECTs at a final concentration of 250 nM. DMSO was used as the vehicle control. ECTs were cultured for 5 weeks under electrical stimulation with mavacamten or DMSO. The medium was replaced twice a week. A contractility assessment in the presence of mavacamten or DMSO was performed 4 weeks after the initiation of treatment. On week 5, the medium was replaced with fresh medium without the test articles and incubated for 24 h before Ca^2+^ transient and contractility measurements.

### 2.12 Statistics

Statistical analyses were performed using Prism version 9 (GraphPad Software, Inc., CA, USA). Data are presented as mean ± SEM except EC_50_ and IC_50_ values, which are presented as the geometric mean. For EC_50_/IC_50_ calculations, the nonlinear fit function of Prism (Sigmoidal, 4 PL) was used to find the best fit for the data. Student’s t-test was used for simple comparisons and One-Way ANOVA followed by Sidak post-hoc test was used for multiple comparisons. Differences at *p* < 0.05 were considered statistically significant. The replicate numbers are indicated in the figure captions.

## 3 Results

### 3.1 Generation, electrical stimulation, and assessment of ECTs

**FIGURE 1:**
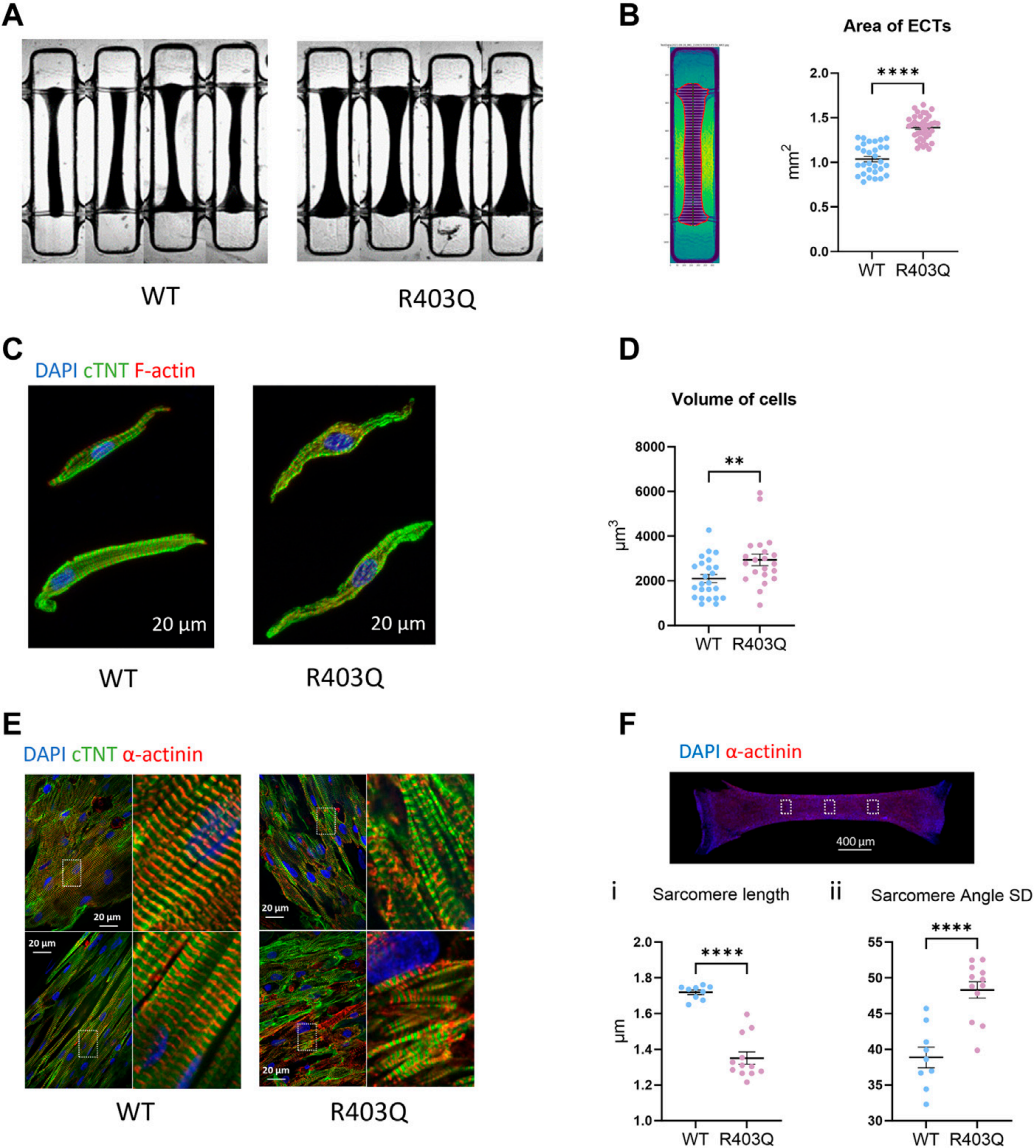
Hypertrophy and sarcomere disorganization in ECTs and individual cardiomyocytes. **(A)** Representative bright field images of ECTs used in area measurements. **(B)** Left: Representative image of an ECT segmented by custom software. Right: Quantification of ECT area (R403Q n = 40 ECTs, WT n = 33 ECTs from 2 seedings). **(C)** Representative confocal images of IF stained isolated cardiomyocytes. **(D)** Quantification of cell volume based on cardiac Troponin-T signal (R403Q n = 21 cells from 3 ECTs, WT n = 24 cells from 3 ECTs). **(E)** Representative confocal images under ×60 magnification from IF stained ECTs. **(F)** *Top:* Representative confocal image under ×2 magnification, boxes denote areas used for sarcomere analysis. *Bottom:* Quantification of (i) sarcomere length and (ii) sarcomere angle standard deviation (SD) of ECTs (R403Q (n = 12 images from 4 ECTs, WT n = 9 images from 3 ECTs; from 2 seedings). Data presented as individual data points with mean (line) ± standard error of the mean. An unpaired, two-tailed t-test was used to determine significance; ** *p* < 0.01; **** *p* < 0.0001.

To create a hypertrophic cardiomyopathy disease model using the Biowire platform, 3D ECTs were generated using a commercially available cell line MYH7-R403Q hiPSC-CMs (R403Q) derived from a HCM patient carrying the mutation and compared to its mutation-corrected isogenic control (WT). A total of 6 batches of R403Q and WT ECTs were generated in parallel from 2 separate iPSC-CM differentiations for phenotypic characterization and drug tests. During the first week of culture, cells seeded in Biowire platform self-organized into 3D ECTs (Figure 1A) and exhibited spontaneous contractions at similar rates in both cell lines. From day 7 of culture, R403Q and WT ECTs were electrically field stimulated for 6 weeks to promote maturation, during which spontaneous contraction gradually stopped in most ECTs (Supplementary Figure S2). At the end of week 7, the electrical stimulation of the ECTs was dropped to a frequency of 3 Hz to stabilize the functional properties of ECTs. Assessments of morphology, function and gene expression were then conducted on ECTs generated in at least 2 separate seedings between week 7 and week 10. The timing of each assessment is individually specified in the following sections.

### 3.2 R403Q ECTs exhibit pathological features of HCM

We first compared the gross morphology of R403Q and WT ECTs generated from 2 seedings in week 7 using brightfield images taken under ×2 magnification. The area of R403Q ECTs (1.39 ± 0.02 mm^2^, n = 30 ECTs) was an average of 1.4-fold larger than WT ECTs (1.04 ± 0.03 mm^2^, n = 30 ECTs; Figures 1A, B). To test if the increase in R403Q ECT area was the result of an increase in cell volume, we enzymatically isolated cells from randomly selected ECTs in week 8, stained the ECTs with cardiac Troponin-T and imaged the cells using confocal microscopy. The 3D cell structure of CMs derived from R403Q ECTs had an average volume of 2,936 ± 258 μm^3^ (n = 21 cells from 3 ECTs), about 1.4-fold larger than those from WT ECTs (2,100 ± 181 μm^3^, n = 24 cells from 3 ECTs), indicating evidence of cellular hypertrophy in the R403Q ECTs (Figures 1C, D; Supplementary Figure S1). Additionally, in isolated cells, sarcomere disorganization was observed in 76% of R403Q CMs (16 out of 21) but not in the WT CMs (Figure 1C). Similar results were obtained from confocal imaging of intact ECTs. In WT ECTs, immunofluorescent staining with α-actinin2 and cardiac Troponin-T antibodies showed well organized sarcomere structure and myofibrils in parallel alignment (Figure 1E Left). In contrast, R403Q ECTs show disorganized sarcomeres and myofibrils (Figure 1E Right). Further analysis using the α-actinin2 signal, a marker of the Z-disc, we quantified the averaged sarcomere length (median distance between 2 adjacent Z-disc in each area) in mature R403Q ECTs to be 1.35 ± 0.03 µm (n = 12 images from 3 ECTs), significantly shorter than that of WT ECTs (1.72 ± 0.01 µm, n = 9 images from 3 ECTs) (Figure 1Fi). The sarcomere organization was quantified as the standard deviation of the angle between 2 adjacent Z-discs. The standard deviation of the angle was significantly greater for R403Q ECTs (48.30 ± 1.15, n = 12 images from 3 ECTs) than for WT ECTs (38.96 ± 1.45, n = 9 images from 3 ECTs), indicating less alignment of sarcomeres in the R403Q ECTs (Figure 1Fii). Taken together, R403Q ECTs generated in the Biowire platform recapitulate hallmarks of HCM, hypertrophy and sarcomere disorganization.

### 3.3 R403Q ECTs demonstrate contractile abnormalities typical of HCM

**FIGURE 2:**
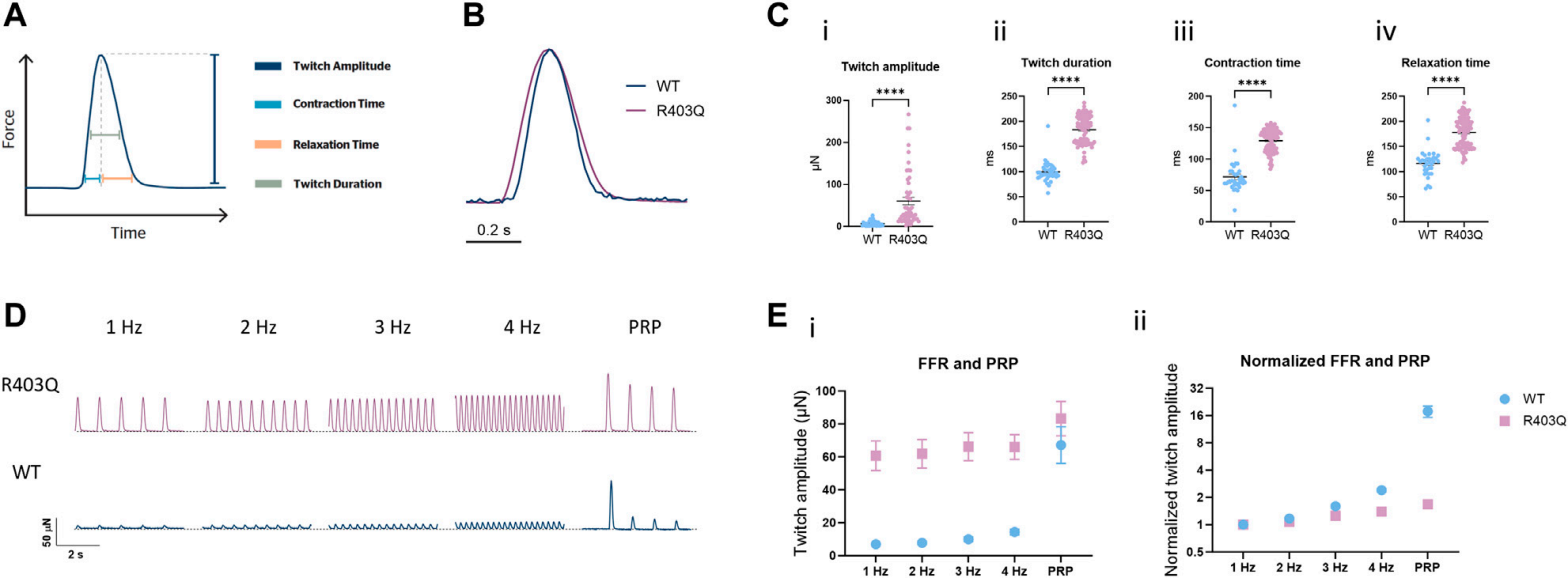
Force-frequency relationship and post-rest potentiation of ECTs. **(A)** Diagram representing the contractility parameters derived from the force vs time curves. **(B)** Overlay of contractile traces normalized to the peak twitch amplitude for week 7 R403Q and WT ECTs at 1 Hz stimulation. **(C)** Baseline contractility comparison of (i) twitch amplitude, (ii) twitch duration, (iii) contraction time, and (iv) relaxation time between WT (n = 33 ECTs) and R403Q (n = 53 ECTs). **(D)** Comparison of representative contractile traces of week 7 R403Q and WT ECTs at stimulation frequencies from 1 to 4 Hz and post-rest potentiation. **(E)** Comparison of (i) raw twitch amplitude and (ii) twitch amplitude normalized to 1 Hz between WT (n = 33 ECTs) and R403Q (n = 53 ECTs). Data were obtained from 2 seedings and presented as individual data points with mean (line) ± standard error of the mean. An unpaired, two-tailed t-test was used to determine significance; **** *p* < 0.0001.

To study the effect of the R403Q mutation on ECT contractile properties, baseline contractility was compared between R403Q (n = 53 ECTs) and its isogenic control (n = 33 ECTs) from 3 seedings in week 7. The contractile parameters were obtained by tracking and fitting the deflection of the polymers wires as previously described (Zhao et al., 2019) (Figure 2A). When stimulated at 1Hz, R403Q ECTs generated a twitch amplitude of 60.7 ± 9.0 µN, 8.6-fold greater than the isogenic control ECTs (7.0 ± 1.0 µN) and had significantly longer twitch durations relative to WT ECTs (peak width at 50% peak height; 183.4 ± 2.6 ms vs 99.4 ± 3.5 ms; Figures 2B, C). The increased twitch duration time for R403Q ECTs is associated with prolongation of both contraction time (time from 10% peak to peak height; 129.2 ± 1.7 ms) and relaxation time (time from peak height to 10% peak, 178.0 ± 2.8 ms) as compared to those of isogenic control (70.2 ± 4.6 ms and 113.8 ± 5.0 ms, respectively; Figure 2C). Notably, similar changes in the contractile properties of R403Q ECTs relative to their isogenic control have been reported for other iPSC-CM-based HCM models (Eschenhagen and Carrier, 2019).

The interaction between the contractile machinery and intracellular Ca^2+^ handling was further assessed by measuring the frequency-dependent regulation of contractility (force-frequency relationship; FFR) and post-rest potentiation (PRP) of ECTs. A positive slope of the FFR and the presence of PRP, which are usually absent in ECTs without electrical stimulation (Zhao et al., 2019), gradually emerged over the first few weeks of the electrical stimulation protocol in WT ECTs. While greater forces were generated at all tested frequencies (1–4 Hz) by R403Q ECTs relative to WT controls, R403Q ECTs had smaller fold-changes in twitch amplitude in response to increasing stimulation frequencies than the isogenic control, especially at 3 and 4 Hz (Figures 2D, E). While R403Q ECTs generated PRP contractions with absolute force values greater than the WT ECTs, this only translates to a force potentiation of 1.7-fold after a rest period (normalized to 1 Hz) for R403Q ECTs vs an 18-fold increase for WT ECTs. These findings are consistent with R403Q ECTs having impaired excitation-contraction coupling relative to WT ECTs.

### 3.4 R403Q ECTs exhibit changes in action potentials and Ca^2+^ handling

**FIGURE 3:**
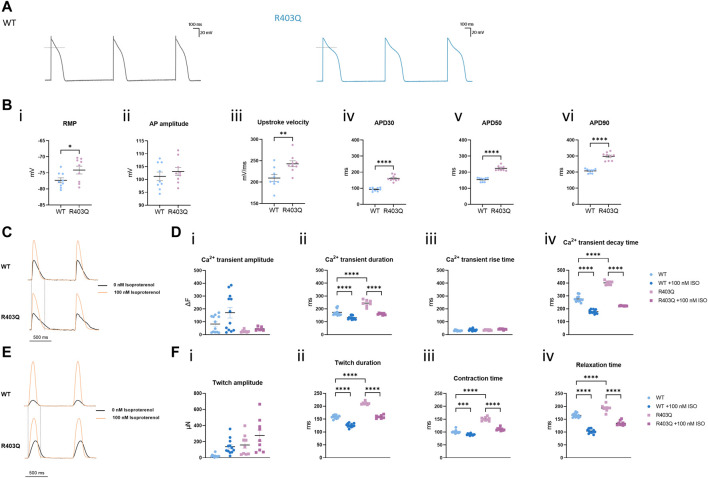
R403Q ECTs exhibit prolonged action potential duration and Ca^2+^ transient decay time. **(A)** Representative action potential traces for R403Q (grey line) and WT (blue line) ECTs. **(B)** Quantification of (i) APD_30_, (ii) APD_50_, (iii) APD_90_, (iv) upstroke velocity, (v) AP amplitude and (vi) RMP for WT (n = 9 cells from 3 ECTs) and R403Q (n = 9 cells from 3 ECTs). **(C)** Overlay of representative Ca^2+^ transient traces from WT (top) and R403Q (bottom) ECTs under 1 Hz stimulation in the absence and presence of 100 nM isoproterenol. **(D)** Quantification of (i) Ca^2+^ transient amplitude, (ii) Ca^2+^ transient duration, (iii) Ca^2+^ transient rise time and (iv) Ca^2+^ transient decay time for WT (n = 12 ECTs) and R403Q (n = 7 ECTs) in the absence and presence of 100 nM isoproterenol. **(E)** Overlay of representative contractile traces from WT (*Top*) and R403Q (*Bottom*) ECTs under 1 Hz stimulation in the absence and presence of 100 nM isoproterenol. **(F)** Quantification of (i) twitch amplitude, (ii) twitch duration, (iii) contraction time and (iv) relaxation time for WT (n = 12 ECTs) and R403Q (n = 9 ECTs) in the absence and presence of 100 nM isoproterenol. Ca^2+^ transient data were obtained from 2 seedings. Data presented as individual data points with mean (line) ± standard error of the mean. In **(C)**, an unpaired, two-tailed t-test was used to determine significance; * *p* < 0.05, ** *p* < 0.01, **** *p* < 0.0001. In **(D)** and **(F)**, a One-Way ANOVA followed by Sidak *post hoc* test was used to determine significance. *** *p* < 0.001, **** *p* < 0.0001.

To investigate the effect of the R403Q mutation on the electrophysiological properties of ECTs, we conducted sharp-electrode action potential (AP) recordings of cardiomyocytes in intact ECTs. R403Q and WT (n = 9 cells from 3 ECTs per line) both show APs with key features of adult-like ventricular cardiomyocytes including a resting membrane potential (RMP) close to −80 mV, a fast upstroke velocity, a positive overshoot, a visible plateau phase and the absence of phase 4 depolarization (Figures 3A, B i-iii). Despite these similarities, R403Q ECTs showed significantly prolonged action potential durations (APDs) as compared to WT ECTs by an average of 68 ms, 70 ms and 91 ms for APD_30_, APD_50_ and APD_90_, respectively (Figure 3Biv–vi).

A similar kinetic change was observed in Ca^2+^ transient measurements from R403 ECTs. R403Q (n = 12 ECTs from 2 seedings) exhibited a significantly wider Ca^2+^ transients as well as a smaller Ca^2+^ transient amplitude than the WT (n = 9 ECTs from 2 seedings) (Figures 3C, D i-ii). Broadening of the Ca^2+^ transient peak for R403Q ECTs can be attributed to the prolongation of the Ca^2+^ transient decay time given that the Ca^2+^ transient rise time was comparable between the R403Q and WT ECTs (Figure 3D iii-iv). Peak broadening is also evident when comparing the contractile measurements of R403Q ECTs to those of the isogenic control (Figures 3E, F). Notably, in response to treatment with 100 nM isoproterenol, a β-adrenergic receptor agonist, the Ca^2+^ transient amplitude of WT ECTs (ΔF = 88 ± 23) increased approximately 5- fold more than in R403Q ECTs (ΔF = 18 ± 2), whereas twitch amplitude increased approximately the same amount for both R403Q and WT ECTs, (117 ± 31 μN and 115 ± 22 μN, respectively). Herein, we observed cardiac dysfunction in R403Q ECTs that can be attributed to all aspects of the excitation-contraction coupling machinery as evidenced by APD prolongation and broadening of both Ca^2+^ transient and contractile peaks. Additionally, we observe an inability of R403Q ECTs to respond to isoproterenol with as robust a calcium response as observed with WT ECTs. These results suggest R403Q ECTs generated in the Biowire platform can recapitulate the complexity of the pathogenic HCM phenotype.

### 3.5 R403Q ECTs show gene expression changes consistent with the HCM phenotype

**FIGURE 4:**
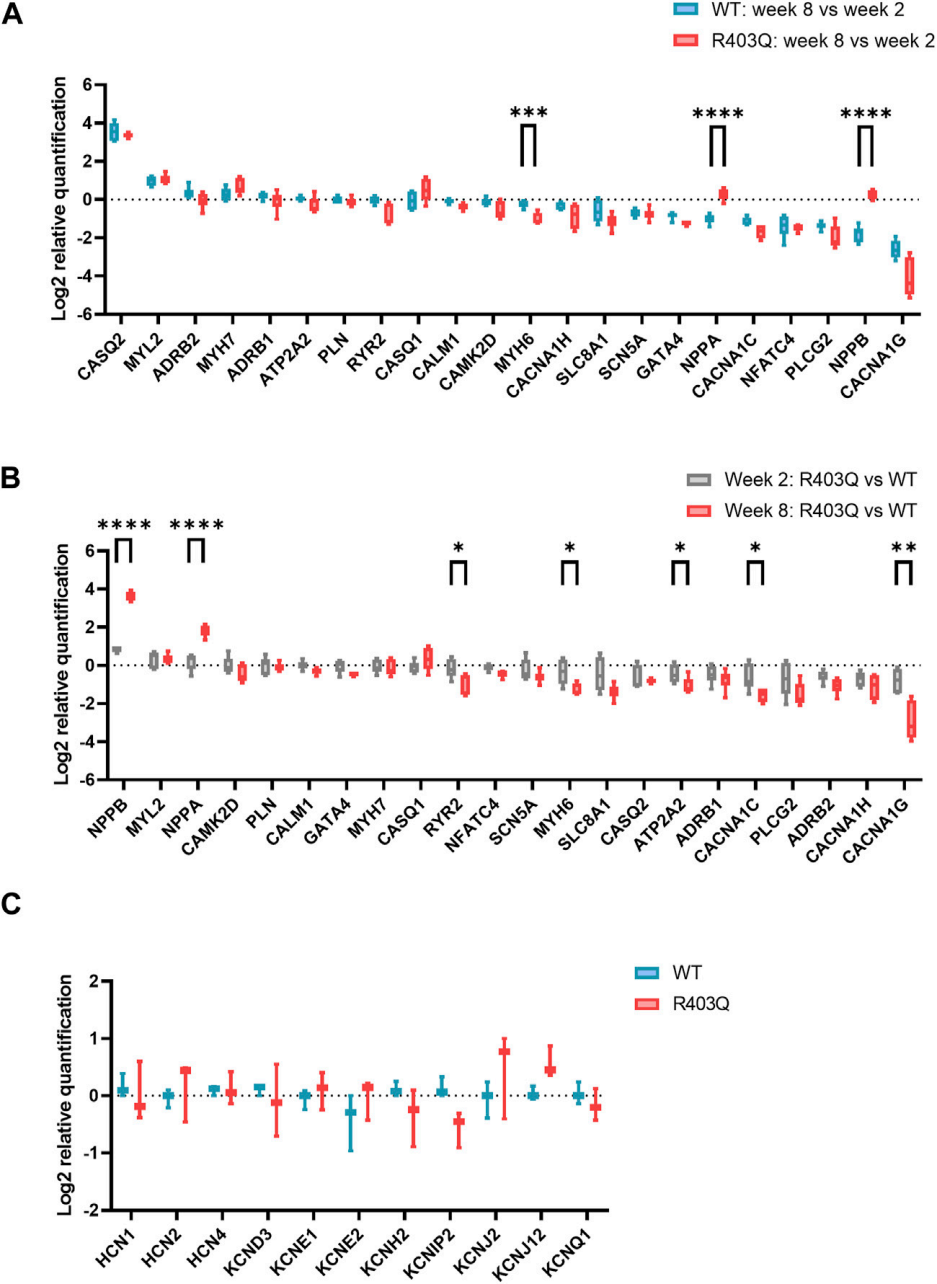
RT-qPCR experiments on R403Q and WT ECTs. **(A)** Relative gene expression of week 8 (n = 6 ECTs) vs week 2 (n = 6 ECTs) R403Q compared to relative gene expression of week 8 (n = 6 ECTs) vs week 2 (n = 6 ECTs) WT. **(B)** Relative gene expression of R403Q vs WT in week 2 (R403Q and WT, n = 6 ECTs) compared to relative gene expression of R403Q vs WT in week 8 (R403Q and WT, n = 6 ECTs). **(C)** Relative K^+^ channel gene expression of R403Q vs WT ECTs in week 8. (R403Q and WT, n = 3 ECTs). Data were obtained from 2 seedings and presented as box and whisker plots with min and max whiskers. An unpaired, two-tailed t-test was used to determine significance; * *p* < 0.05, ** *p* < 0.01, *** *p* < 0.001, **** *p* < 0.0001.

To investigate the potential molecular mechanisms underpinning the observed functional differences between R403Q and WT ECTs, relative quantification of RT-qPCR was performed using RNA isolated from week 2 and week 8 ECTs. In the present study, we focused on genes that encode for cardiac ion channels (*CACNA1C*, *CACNA1H, CANNA1G, SCN5A, HCN1, HCN2, HCN4, KCND3, KCNE1, KCNE2, KCNH2, KCNIP2, KCNJ2, KCNJ12* and *KCNQ1*), Ca^2+^ signaling pathway proteins (*ADRB1, ADRB2, PLCG2, ATP2T2, PLN, RYR2, CASQ1, CASQ2, CALM1* and *CAMK2D*) and HCM related genes (*MYH7, MYH6, MYL2, GATA4*, and *NFATC4*). From week 2 to week 8, both WT and R403Q (n = 6 ECTs per line per time points from 2 seedings) showed upregulation of *CASQ2, MYL2* and downregulation of *CACNA1C, NFATC4, PLCG2* and *CACNA1G* to the same extent. However, *NPPA* and *NPPB* were downregulated by approximately 2- and 3-fold, respectively, in week 8 vs week 2 WT ECTs, while no change in expression levels were observed for R403Q ECTs (Figure 4A). Notably, the differences in gene expression between the R403Q and WT ECTs is more pronounced at week 8 than at week 2 (Figure 4B). For instance, R403Q ECTs showed an ∼2-fold increase in *NPPB* expression compared to WT ECTs at week 2 but an ∼ 13-fold increase by week 8. Also, *RYR2*, *MYH6, ATP2A2, CACNA1C, CACNA1H* and *CACNA1G* showed a 2-fold or greater decrease in expression in R403Q ECTs relative to WT ECTs at week 8 but not at week 2. In contrast, we did not find any significant difference in gene expression of key cardiac potassium channels between R403Q and WT at week 8 (Figure 4C).

### 3.6 WT and R403Q ECTs respond similarly to inotropes in acute contractility experiments

**FIGURE 5:**
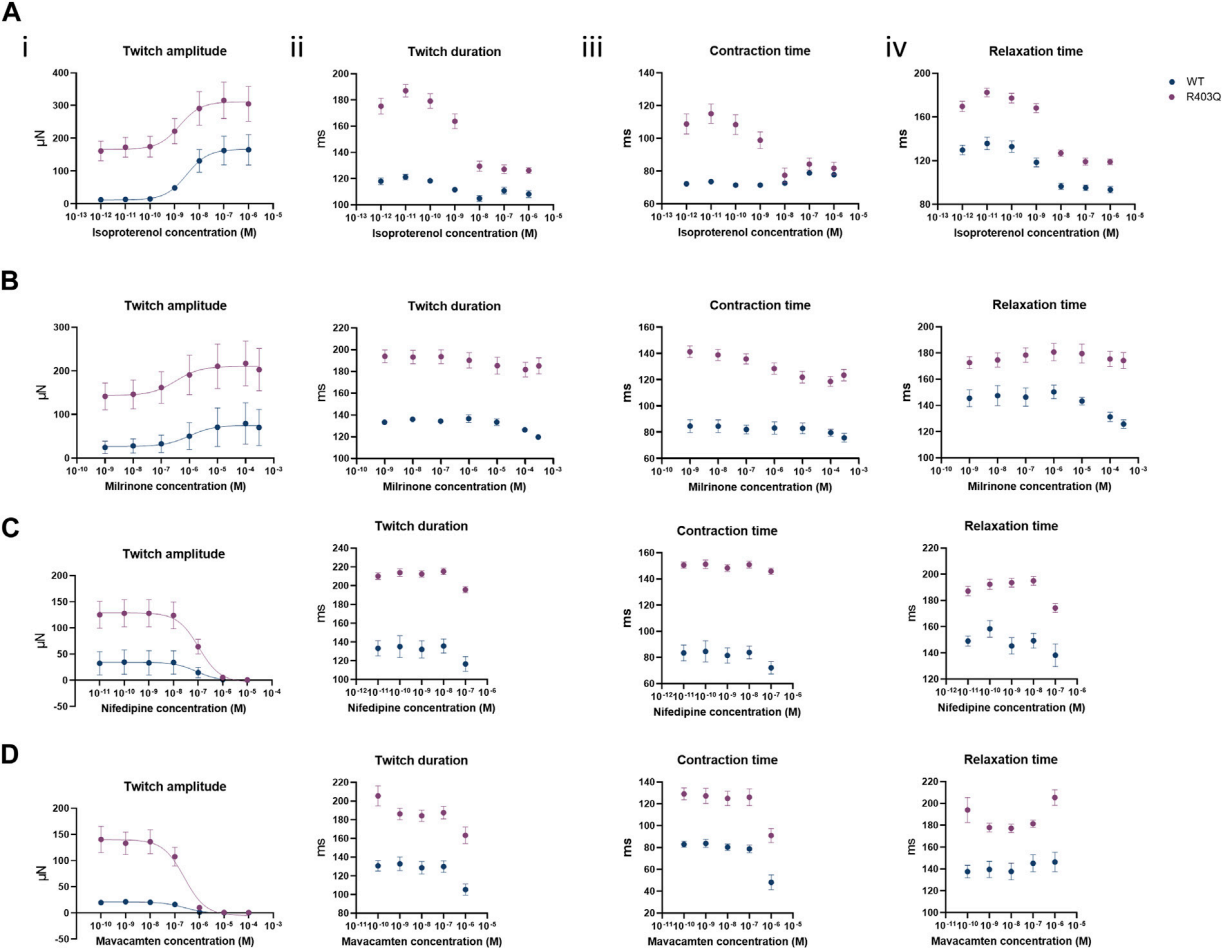
Acute contractility measurements of ECTs in response to inotropes. **(A)** Quantification of (i) twitch amplitude, (ii) twitch duration, (iii) contraction time and (iv) relaxation time in response to increasing concentrations of isoproterenol (R403Q n = 10 ECTs and WT n = 5 ECTs from 2 seedings). **(B)** Quantification of (i) twitch amplitude, (ii) twitch duration, (iii) contraction time and (iv) relaxation time in response to milrinone (R403Q n = 5 ECTs and WT n = 2 ECTs from 2 seedings). **(C)** Quantification of (i) twitch amplitude, (ii) twitch duration, (iii) contraction time and (iv) relaxation time in response to nifedipine (R403Q n = 6 ECTs and WT n = 4 ECTs from 2 seedings). **(D)** Quantification of (i) twitch amplitude, (ii) twitch duration, (iii) contraction time and (iv) relaxation time in response to mavacamten (R403Q n = 8 ECTs and WT n = 4 ECTs from 2 seedings). Data presented as individual data points with mean (line) ± standard error of the mean. Curves were fitted using four parameter logistic regression. Time constants for nifedipine and mavacamten are shown to the concentrations where twitch amplitudes are greater than zero.

To assess the effect of the R403Q mutation on the functionality of pathways that modulate cardiac contractility, ECTs were exposed to agents known to affect the contractility of the human myocardium. The concentration-dependent contractility response to positive and negative inotropes was compared between R403Q and WT ECTs generated from 2 seedings (Figure 5; Table 2). When treated with isoproterenol, R403Q (n = 10 ECTs) and WT (n = 5 ECTs) both reached the maximal increase in contractile force at 100 nM (Figure 5Ai). While isoproterenol shortened twitch duration in both R403Q and WT ECTs, a larger decrease in twitch duration in response to 100 nM isoproterenol was observed for R403Q (decrease of 48 ± 3 ms vs 7 ± 4 ms in WT). Furthermore, while 100 nM isoproterenol similarly shortened relaxation time in both R403Q and WT ECTs (R403Q 51 ± 3 ms, WT 34 ± 4 ms), it only shortened contraction time in R403Q ECTs (decrease of 30 ± 3 ms vs increase of 7 ± 2 ms in WT). Both R403Q (n = 5 ECTs) and WT (n = 2 ECTs) responded similarly to the PDE3 inhibitor, milrinone, with a maximal contractile force increase at 100 µM of 75 ± 22 μN and 55 ± 33 μN, respectively (Figure 5B). Both the R403Q and WT ECTs responded to isoproterenol and milrinone with a similar EC_50_ (Table 2).

**TABLE 2:** Summary of concentration-response curve fits for inotropes in acute contractility assays.

		WT	R403Q
isoproterenol	EC_50_	2.5 nM	1.6 nM
	Hill coefficient	1.0	0.8
	n (ECTs)	5	10
milrinone	EC_50_	1.3 µM	0.4 µM
	Hill coefficient	0.7	0.6
	n (ECTs)	2	5
nifedipine	IC_50_	72.6 nM	99.4 nM
	Hill coefficient	−1.7	−1.4
	n (ECTs)	4	6
mavacamten	IC_50_	246 nM	247 nM
	Hill coefficient	−1.7	−1.6
	n (ECTs)	4	8

As for negative inotropes, both the L-type voltage-gated Ca^2+^ channel blocker, nifedipine, and the cardiac myosin ATPase inhibitor, mavacamten, completely inhibited ECT contraction at high concentrations (>1 µM and >10 μM, respectively) and both R403Q and WT ECTs responded to nifedipine and mavacamten with a similar IC_50_ (Figures 5C, D; Table 2). These results suggest both WT and R403Q ECTs can respond to inotropes with known effects in acute contractility experiments as reported previously (Feric et al., 2019) but do not distinguish the pathological phenotype.

### 3.7 Chronic mavacamten of R403Q ECTs recapitulates many hallmarks of HCM treatment

**FIGURE 6:**
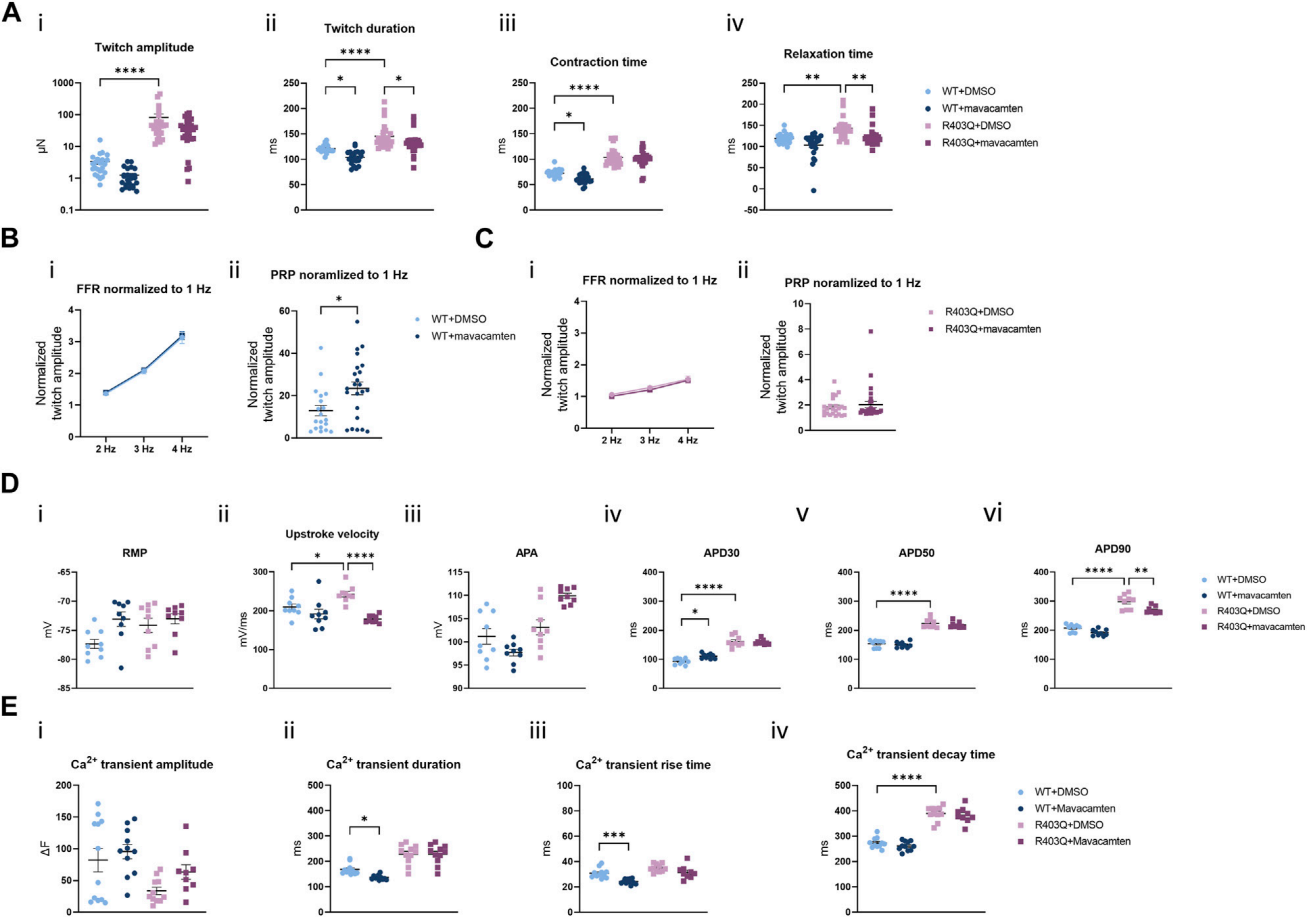
Functional impact of chronic mavacamten treatment on R403Q and WT ECTs. **(A)** Quantification of (i) twitch amplitude, (ii) twitch duration, (iii) contraction time and (iv) relaxation time for ECTs chronically treated with 250 nM mavacamten (WT n = 28 ECTs, R403Q n = 23 ECTs) or DMSO (WT n = 24 ECTs, R403Q n = 24 ECTs). **(B)** Twitch amplitude at 2, 3 and 4 Hz (i) and PRP (ii) normalized to 1 Hz for WT ECTs chronically treated with 250 mM mavacamten (n = 23 ECTs) or DMSO (n = 19 ECTs). **(C)** Twitch amplitude at 2, 3 and 4 Hz (i) and PRP (ii) normalized to 1 Hz for R403Q ECTs chronically treated with 250 mM mavacamten (n = 28 ECTs) or DMSO (n = 23 ECTs). **(D)** Quantification of (i) RMP, (ii) upstroke velocity, (iii) AP amplitude, (iv) APD_30_, (v) APD_50_ and (vi) APD_90_ between WT and R403Q ECTs chronically treated with 250 mM mavacamten or DMSO (n = 9 cells from 3 ECTs for each group). **(E)** Quantification of Ca^2+^ transients (i) amplitude, (ii) duration, (iii) rise time and (iv) decay time for ECTs chronically treated with 250 mM mavacamten (WT n = 11 ECTs and R403Q n = 9 ECTs) or DMSO (WT n = 12 ECTs and R403Q n = 11 ECTs). Data were obtained from 2 seedings and presented as individual data points with mean (line) ± standard error of the mean. In **(A)**, **(D)** and **(E)**, a One-Way ANOVA followed by Sidak *post hoc* test was used to determine significance; in **(B)** and **(C)**, an unpaired, two-tailed t-test was used to determine significance; * *p* < 0.05, ** *p* < 0.01, *** *p* < 0.001 and **** *p* < 0.0001.

Mavacamten is a first-in-class FDA-approved myosin ATPase inhibitor for HCM treatment (Green et al., 2016; Ho et al., 2020; Olivotto et al., 2020). In our acute contractility experiments we were only able to observe the negative inotropic effects of mavacamten shared by many other drugs like L-type calcium channel blockers. Therefore, we investigated the effect of chronic treatment with mavacamten on ECTs to better mimic the effects of the HCM treatment *in vitro*. ECTs were cultured for up to 8 weeks in Induction 3 medium with 250 nM mavacamten, at which concentration ECT twitch amplitude was inhibited by about 50% in the acute contractility experiment (Figure 6A; Table 2). To avoid affecting initial ECT formation, mavacamten treatment was initiated 12 days after ECT generation. For the vehicle control group, a matched concentration of DMSO was added to the culture medium. The effect of chronic incubation on ECT contractility was first assessed at week 7 in the presence of mavacamten (WT n = 28 ECTs, R403Q n = 23 ECTs) or DMSO (WT n = 24 ECTs, R403Q n = 24 ECTs) in 2 separate seedings. We observed a reduction in twitch amplitude by 63% in WT ECTs (1.3 ± 0.2 µN) and by ∼50% in R403Q ECTs (41.7 ± 5.9 µN) compared to their respective DMSO control groups (WT 3.4 ± 0.7 µN, R403Q 82.4 ± 23.4 µN) (Figure 6Ai). In addition, twitch duration was also reduced by 13.4 ms on average in mavacamten-treated R403Q ECTs and 16.8 ms on average in mavacamten-treated WT ECTs compared to their DMSO controls (Figure 6Aii). Chronic mavacamten treatment also shortened contraction and relaxation time in WT ECTs by an average of 11.3 ms and 15.9 ms, respectively, relative to the DMSO controls, whereas in R403Q ECTs only relaxation time was shortened (by 20.9 ms on average) relative to the control group (Figure 6Aiii, iv). Contraction time in R403Q ECTs was unaffected by chronic mavacamten treatment when compared with the DMSO control. No effect on FFR was observed in either WT or R403Q ECTs following chronic mavacamten treatment relative to the respective DMSO control groups. However, an ∼2-fold increase in PRP was observed in mavacamten-treated WT ECTs relative to the DMSO control, whereas no effect of mavacamten treatment was observed in the PRP of R403Q ECTs relative to the control group (Figures 6B, C).

The effect of chronic treatment by 250 nM mavacamten on AP recordings (Figure 6D) and Ca^2+^ transient measurements (Figure 6E) was also determined. Measurements were taken 24 h after the removal of the test articles, mavacamten or DMSO. We observed on average a 26% reduction in upstroke velocity and a 29.2 ms reduction in APD_90_ in mavacamten-treated R403Q ECTs relative to the DMSO control (n = 9 cells from 3 ECTs in both conditions) (Figure 6Dii, vi). In contrast, we observed that mavacamten-treated WT ECTs had increased APD_30_ compared to DMSO controls (by 17.8 ms on average, n = 9 cells from 3 ECTs in both conditions) (Figure 6Div). Ca^2+^ transient duration and Ca^+^ transient rise time decreased by an average of 31.6 ms and 6.6 ms respectively in mavacamten-treated WT ECTs (n = 11 ECTs) relative to DMSO controls (n = 12 ECTs), whereas no significant difference was found between the mavacamten- and DMSO-treated R403Q ECTs (n = 9 and 11 ECTs, respectively) (Figure 6E). The shortening of relaxation time and APD_90_ in the mavacamten-treated R403Q ECTs suggests the reversal of some aspects of the HCM disease phenotype with mavacamten treatment.

**FIGURE 7:**
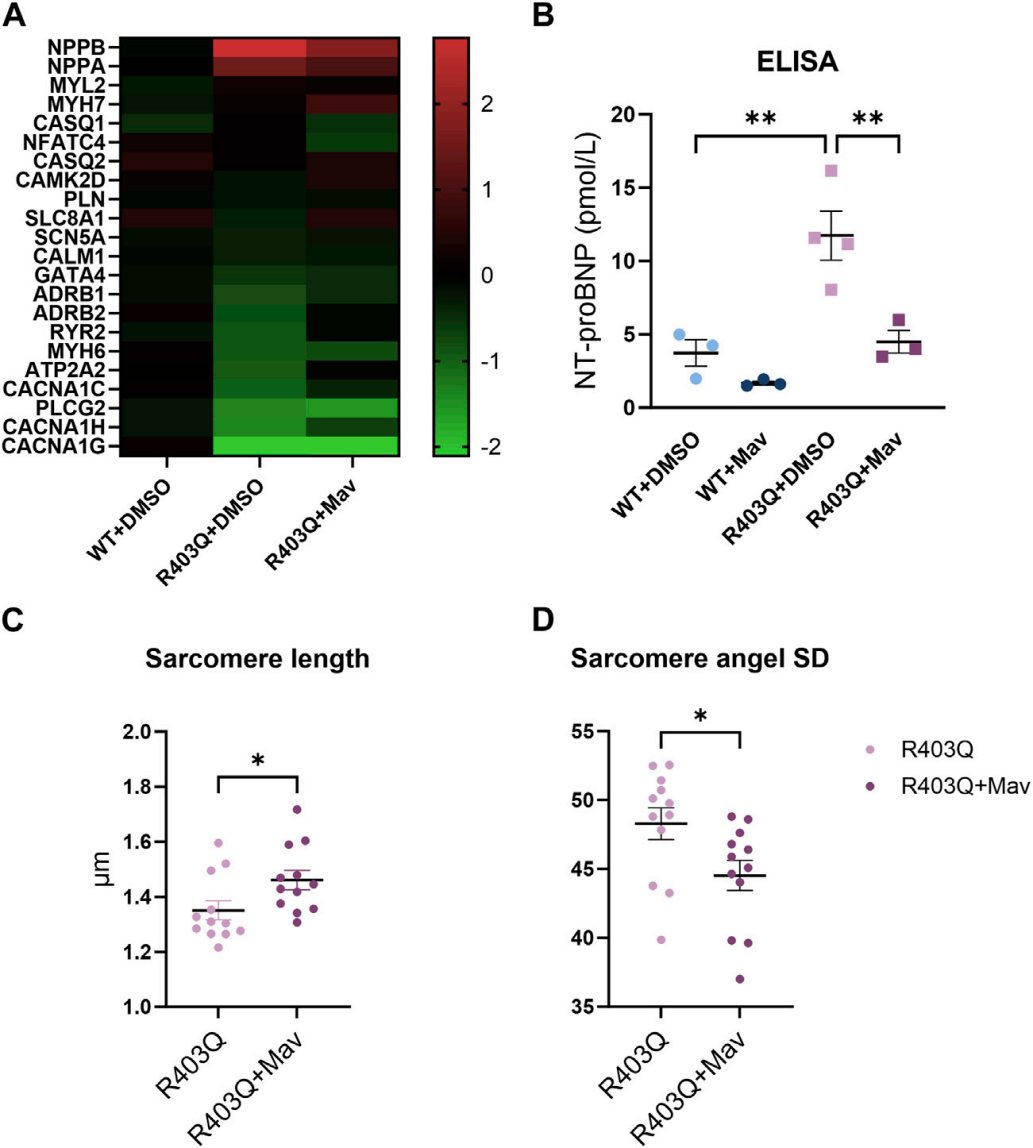
Changes in gene expression and sarcomere organization in mavacamten-treated R403Q and WT ECTs. **(A)** Heatmap of log2(fold-change) of relative gene expression in DMSO-treated and 250 nM mavacamten-treated R403Q ECTs compared to DMSO-treated WT ECTs. Means of 6 individual ECTs of each group are presented. Genes are sorted in descending order of fold-change in abundance in DMSO-treated R403Q ECTs. **(B)** NT-proBNP concentration in conditioned medium from WT and R403Q ECTs treated with 250 nM mavacamten (WT and R403Q, both n = 3 biological replicants) and DMSO (WT n = 3 and R403Q n = 4 biological replicants). Quantification of **(C)** sarcomere length and **(D)** sarcomere angle standard deviation from DMSO-treated (n = 12 images from 4 ECTs) or mavacamten-treated R403Q ECTs (n = 12 images from 4 ECTs). Data were obtained from 2 seedings and presented as individual data points with mean (line) ± standard error of the mean. A One-Way ANOVA followed by Sidak *post hoc* test was used to determine significance in **(B)**. An unpaired, two-tailed t-test was used to determine significance in **(C,D)**. * *p* < 0.05, ** *p* < 0.01.

To investigate the effect of chronic mavacamten treatment on transcriptional regulation, we performed RT-qPCR experiments comparing the gene expression of DMSO-treated WT ECTs, and DMSO- or mavacamten-treated R403Q ECTs (n = 6 ECTs per line per condition). The RT-qPCR results demonstrate that many transcriptional changes attributable to the MYH7-R403Q mutation were partially or fully reversed by mavacamten treatment (Figure 7A and Supplementary Figure S3). Partial reversal of transcriptional change was observed in the expression of *NPPA*, *NPPB* and *CANCA1C*. For example, compared to DMSO-treated WT ECTs, the *NPPB* expression in DMSO-treated R403Q ECTs was upregulated by ∼7-fold, which was reduced by half in mavacamten treated R403Q ECTs. A full reversal of the transcriptional changes in mavacamten treated R403Q ECTs was observed in the expression of *ADRB2*, *ATP2A2* and *RYR2*. Notably, the *MYH7*/*MYH6* ratio was 0.9 ± 0.1 in DMSO-treated WT ECTs and 2.1 ± 0.1 in DMSO-treated R403Q ECTs. This finding is consistent with the increase in the *MYH7*/*MYH6* ratio reported for hypertrophied human heart (Lowes et al., 1997) and other iPSC-CMs carrying HCM-associated mutations (Mosqueira et al., 2018; Chai et al., 2023). No significant difference in the gene expression of key cardiac potassium channels was observed between mavacamten- and DMSO-treated ECTs (Supplementary Figure S4).

To further explore the *NPPB* findings, we measured N-terminal B-type natriuretic peptide (NT-proBNP) levels in the conditioned medium using an ELISA assay. BNP and its precursor NT-proBNP are the cardiac biomarkers encoded by the *NPPB* gene. Expression level of BNP or NT-proBNP in CMs and plasma serves as a biomarker for myocardial hypertrophy, stress, or failure (Seferian et al., 2007; Man et al., 2018). The NT-proBNP concentration of the R403Q ECT conditioned medium (n = 4 samples) was 3-fold higher than for the WT ECTs (n = 3 samples) (Figure 7B). While chronic treatment with mavacamten reduced the NT-proBNP concentration in the conditioned medium of both R403Q and WT ECTs (both n = 3 samples), the treatment decreased the NT-proBNP concentration of R403Q ECTs to that of WT ECTs treated with DMSO. These results suggest the reversal of the elevated BNP levels, a diagnostic indicator of HCM disease prognosis and progression, with chronic mavacamten treatment.

Lastly, we observed that chronic mavacamten treatment partially reversed the changes in sarcomere structure caused by the R403Q mutation. The average sarcomere length in R403Q ECTs treated with 250 nM mavacamten (1.46 ± 0.04 µm, n = 12 images from 4 ECTs) was significantly longer than for DMSO-treated R403Q ECTs (1.35 ± 0.03 µm, n = 12 images from 4 ECTs) (Figure 7C). Similarly, the sarcomere angle standard deviation significantly decreased from 48.30 ± 1.15 in DMSO-treated R403Q ECTs to 44.53 ± 1.10 in mavacamten-treated R403Q ECTs (Figure 7D).

## 4 Discussion

Despite major advances enabling better identification and classification of HCM patients over the last 60 years, therapeutic options either to manage the risk of life-threatening arrhythmic events or to prevent adverse disease progression remain limited for these patient populations (Maron, 2018; Maron et al., 2022a). A disease modeling system that faithfully recapitulates the clinical phenotype of HCM is crucial to the development of novel mechanism-driven therapies. Although transgenic animal models enable detailed pathophysiological and molecular assessments of HCM-related genetic variants, they can have limited relevance to human disease. For instance, the major motor protein that produces contractile force in the sarcomere is coded by *MYH6* in mice but by *MYH7* in humans (Jiang et al., 2013). In addition, LV septum hypertrophy, a hallmark feature of human HCM, is rarely present in mouse models (Duncker et al., 2015), and significant differences in the molecular phenotype of mouse models and HCM patients have been reported (Vakrou et al., 2021).

Cardiomyocytes differentiated from patient-specific iPSCs and the genetically edited isogenic controls have been shown to provide valuable insights into the mechanisms of inherited cardiac diseases (Eschenhagen and Carrier, 2019; Ramachandra et al., 2021). However, disease models using 2D cultured iPSC-CMs present some clear limitations in terms of morphology, contractility, and electrophysiology, resembling human cardiomyocytes at the embryonic stage rather than adult (Sewanan et al., 2020). In 2D culture, iPSC-CMs carrying HCM mutations are smaller than adult ventricular cardiomyocytes (cell volume between 30,000 μm^3^ and 50,000 μm^3^) (Gerdes et al., 1992; Tracy and Sander, 2011; Mollova et al., 2013) with cell volumes ranging from 5.8 µm^3^ to 120 μm^3^ (Wang et al., 2018; Mosqueira et al., 2019). 2D-cultured iPSC-CMs are also variable in shape and lack clear sarcomere organization or alignment (Veerman et al., 2015; Jiang et al., 2018). Conversely, here we report 3D ECTs with a median cell volume of 1887 μm^3^ in WT ECTs and 2,780 μm^3^ in R403Q ECTs, while still smaller than human adult CMs they are much larger than reported for 2D and closer to the cell volumes reported for 0–1-year-old human heart CMs of ∼6,000 μm^3^ (Mollova et al., 2013). The isolated CMs from the ECTs were also over 90% rod shaped and had uniform sarcomere alignment (Figure 1E). Finally, as previously reported, our ECTs have adult-like contractile and electrophysiological responses (Feric et al., 2019). Taken together, Biowire ECTs recapitulate more features of the adult myocardium and are therefore better positioned to model the key features of HCM, when compared to 2D hiPSC-CMs.

Cardiac contractility is a consequence of a precise series of events known as excitation-contraction coupling. This highly orchestrated process links the action potential and changes in the cytoplasmic Ca^2+^ concentration to muscle contraction and relaxation. Physiological changes in HCM seen clinically include increased myocardial contractility and impaired relaxation (Lopes et al., 2013). A prevailing hypothesis suggests HCM-related mutations cause conformational changes in sarcomeric proteins and shift the myosin molecules from the super relaxed state (SRX) toward the actin-bound state, the effect of which is increased myocyte contractility and ATP utilization. In response to the contractile changes, stress-sensing signaling pathways are activated, which induce molecular and histological changes in the myocardium (McNamara et al., 2016; Sarkar et al., 2020). In the present study, we reported hypercontractility along with prolongation of contraction and relaxation time in R403Q ECTs compared to the isogenic control (Figure 2), results consistent with other *in vitro* studies of human iPSC-CMs with *MYH7, MYBPC3, ACTN2*, or *ACTC1* mutations (Eschenhagen and Carrier, 2019). Additionally, the FFR and PRP are often impaired in patients with cardiomyopathy or heart failure (Endoh, 2004). Notably, the ability to measure the FFR and PRP in *in vitro* systems has not been reported by many (Nunes et al., 2013; Ronaldson-Bouchard et al., 2018; Feric et al., 2019; Lu et al., 2021). Herein, we report compromised FFR and PRP properties in R403Q ECTs (Figure 2) in agreement with the observed clinical presentations.

Altered ion channel function and expression, a pathogenic mechanism independent of contractile dysfunction, was reported in several studies using surgical samples from HCM patients and transgenic animal models (Barajas-Martínez et al., 2013; Coppini et al., 2013; Coppini et al., 2018; Lan et al., 2013). Patch clamp recordings from individual cells showed increased L-Type Ca^2+^ current and late Na^+^ current in isolated HCM cardiomyocytes and reduced inward-rectifier K^+^ current, transient outward K^+^ current and delayed rectifier K^+^ currents. These changes are considered as underlying the APD prolongation observed in those samples. Other studies in human *in vitro* (Messer et al., 2016; Smith et al., 2018; Wang et al., 2018) and mouse models (Knollmann et al., 2003; Stöhr et al., 2013) have demonstrated that an increased sensitivity of myofilaments to Ca^2+^ associates with many HCM-related mutations. It is hypothesized that increased Ca^2+^ sensitivity along with altered Ca^2+^ handling properties prolong Ca^2+^ transient decay time, contributing to clinical phenotypes, including diastolic dysfunction and arrythmias, in HCM patients (Santini et al., 2021). In the present study, we demonstrate a reduced Ca^2+^ transient amplitude paired with an increased twitch amplitude in R403Q ECTs compared with WT ECTs, a finding in accordance with an increased Ca^2+^ sensitivity in HCM (Figures 3C–F). We also observed a prolongation of the Ca^2+^ transient decay time and downregulation of Ca^2+^ handling related genes in R403Q ECTs (∼2-fold decrease in *ATP2A2, CACNA1C, CACNA1H* and *CACNA1G*, Figure 4). While we did not observe significant changes in K^+^ channel genes at the transcription level (Figure 4C), a significant APD prolongation was recorded in R403Q ECTs relative to WT ECTs (Figures 3A, B). These results suggest our model of HCM can recapitulate pathological changes of electrophysiology in patient samples and is consistent with the hypothesis of increased Ca^2+^ sensitivity and impaired Ca^2+^ handling.

Current pharmacological management of HCM, such as β-blockers and calcium channel blockers, does not target the pathogenic mechanism of HCM and thus offers little to prevent disease progression (Maron et al., 2022b). The one exception is mavacamten, a recently discovered myosin ATPase inhibitor that directly targets hypercontractility. It was first demonstrated in HCM mice models that mavacamten inhibited contractility of the heart after 2 weeks of oral administration and reduced LV wall thickness after 4 weeks. When administered to young pre-HCM mice, mavacamten also suppressed the development myocyte disarray, myocardial fibrosis and normalized hypertrophic and profibrotic gene expression (Green et al., 2016). In a recent phase III clinic trial, treatment of obstructive HCM with mavacamten for 30 weeks improved exercise capability, New York Heart Association functional class, peak oxygen consumption and outflow tract obstruction (Olivotto et al., 2020). A significant reduction in NT-proBNP plasma level after 4 weeks of mavacamten treatment was reported in another clinic trial for non-obstructive HCM, suggesting improvements in myocardial wall stress (Ho et al., 2020). The treatment effect of mavacamten was also shown in hiPSC-CMs carrying HCM-causing mutations in *MYH7* and *TPM1* genes, including decreased contractility and relaxation time, and reversal of increased cell surface area and BNP expression after 1–4 days of treatment (Wijnker and van der Velden, 2020; Sewanan et al., 2021; Riaz et al., 2022).

We treated ECTs for 5 weeks with mavacamten to provide insights into long-term therapeutic outcomes and to better mimic the treatment regimen used for animal models and patients. We found many HCM phenotypes observed in R403Q ECTs fully or partially reversed with chronic mavacamten treatment, consistent with the findings in the aforementioned studies. Firstly, chronic mavacamten treatment of R403Q ECTs improved the contractile relaxation time and APD_90_ (Figures 6A, D). Secondly, the upregulation of *NPPA* and *NPPB* and downregulation of *ADRB2*, *ATP2A2*, *RYR2* and *CACNA1C* in R403Q ECTs were reverted toward WT levels by chronic mavacamten treatment (Figures 7A, B). Notably, some gene expression differences between WT and R403Q ECTs were only observed at the end of the electrical stimulation protocol (week 8) or were not as pronounced at week 2, i.e., pre-stimulation. These findings suggest that the adult-like morphology, contractility, and electrophysiology of week 8 ECTs might be a pre-requisite for modeling certain aspects of the HCM phenotype. Lastly, we found chronic mavacamten treatment partially reversed the reduced sarcomere length and disorganized sarcomere structure observed for R403Q ECTs (Figures 7C, D), consistent with animal models (Green et al., 2016).

In conclusion, we demonstrate the ability to model key hallmarks of HCM using a patient-derived hiPSC-CM cell line and the Biowire platform. We generated ECTs with an adult-like phenotype, which enabled us to recapitulate features of the disease not evident in pre-stimulation ECTs. In addition to changes in morphology, contractility, Ca^2+^ transients, electrophysiology and gene expression that agree with reports by others from *in vitro* and patient data, we were able to report on FFR and PRP in the disease state, findings that align with clinical observations. Finally, we were able to conduct an extended (5-week) mavacamten treatment at a fraction of the cost of an animal model with similar results as were obtained from animal models and clinical trials. Taken together, these results demonstrate our ability to model the complex disease of HCM in the Biowire platform and provide evidence for both the translational value of our model and the utility of our HCM model in drug discovery.

## Data Availability

The original contributions presented in the study are included in the article/Supplementary Material, further inquiries can be directed to the corresponding author.

## Abbreviations

AP, action potential; APD, action potential duration; BNP, B-type natriuretic peptide; CM, cardiomyocyte; ECT, engineered cardiac tissue; FFR, force-frequency relationship; HCM, hypertrophic cardiomyopathy; hiPSC, human induced pluripotent stem cell; PRP, post-rest potentiation; WT, wild-type.
