# Supplementary material for: Human engineered cardiac tissue model of hypertrophic cardiomyopathy recapitulates key hallmarks of the disease and the effect of chronic mavacamten treatment
Source: Front Bioeng Biotechnol. 2023 Sep 8;11:1227184. doi: 10.3389/fbioe.2023.1227184 (PMC10523579; doi:10.3389/fbioe.2023.1227184)
Supplement: Supplementary file 2 [file Table1.DOCX]

Supplementary Material

**Human Engineered Cardiac Tissue Model of Hypertrophic Cardiomyopathy Recapitulates Key Hallmarks of The Disease and The Effect of Chronic Mavacamten Treatment**

**Kai Wang^1*^, Brian Schriver^1^, Roozbeh Aschar-Sobbi^1^, Alex Yi^1^, Nicole Feric^1^, Michael P. Graziano^1^**

^1^Valo Health, Inc., Department of Tissue Engineering, New York, NY, USA

*** Correspondence:** Kai Wang: kwang@valohealth.com

# Supplementary Figures and Tables

## 1.1 Supplementary Figures

**Supplementary figure 1**. Sarcomere analysis. Representative confocal images showing α-actinin2 signal under 60x magnification from IF stained A) WT and B) R403Q ECTs used for sarcomere analysis. The (i) Z-discs (ii) sarcomeres and (iii) myofibrils are color coded to denote an association. The zoom-in views of the area inside the box are displayed under the original images.

**Supplementary figure 2**. Spontaneous beating rate in R403Q and WT ECTs during 7 weeks of culture

**Supplementary figure 3**. Relative K^+^ channel gene expression for 250 nM mavacamten-treated ECTs compared to DMSO-treated ECTs. Data are presented as box and whisker plots with min and max whiskers (n=3 ECTs for each group).

## 1.2 Supplementary Tables

| **Gene name** | **ThermoFisher assay ID** |
| --- | --- |
| *ADRB1* | Hs02330048_s1 |
| *ADRB2* | Hs00240532_s1 |
| *ATP2A2* | Hs00544877_m1 |
| *CACNA1C* | Hs00167681_m1 |
| *CACNA1G* | Hs00367969_m1 |
| *CACNA1H* | Hs01103527_m1 |
| *CALM1* | Hs00237233_m1 |
| *CAMK2D* | Hs00943554_m1 |
| *CASQ1* | Hs00154281_m1 |
| *CASQ2* | Hs00154286_m1 |
| *GAPDH* | Hs02786624_g1 |
| *GATA4* | Hs00171403_m1 |
| *KCND3* | Hs00542597_m1 |
| *KCNE1* | Hs00264799_s1 |
| *KCNE2* | Hs00270822_s1 |
| *KCNH2* | Hs04234270_g1 |
| *KCNIP2* | Hs01552688_g1 |
| *KCNJ12* | Hs05015288_s1 |
| *KCNJ2* | Hs00265315_m1 |
| *KCNQ1* | Hs00923522_m1 |
| *MYH6* | Hs01101425_m1 |
| *MYH7* | Hs01110632_m1 |
| *MYL2* | Hs00166405_m1 |
| *NFATC4* | Hs00190037_m1 |
| *NPPA* | Hs00383230_g1 |
| *NPPB* | Hs00173590_m1 |
| *PLCG2* | Hs01101857_m1 |
| *PLN* | Hs01848144_s1 |
| *RPL13A* | Hs04194366_g1 |
| *RYR2* | Hs00181461_m1 |
| *SCN5A* | Hs00165693_m1 |
| *SLC8A1* | Hs01062258_m1 |

**Supplementary Table 1** TaqMan probe list for real time qPCR

## 1.3 Supplementary videos

**Supplementary Video 1. 3D view of a representative cardiomyocyte isolated from a mature R403Q Biowire ECT.** Green: cardiac Troponin-T, Blue: DAPI.

**Supplementary Video 2. Representative Ca^2+^ transients recording from an untreated Biowire ECT.**

**Supplementary Video 3. Representative Ca^2+^ transients recording for the same Biowire ECT in Video S2 treated with 100 nM isoproterenol.**
