# Supplementary figures and images for: Human engineered cardiac tissue model of hypertrophic cardiomyopathy recapitulates key hallmarks of the disease and the effect of chronic mavacamten treatment
Source: Front Bioeng Biotechnol. 2023 Sep 8;11:1227184. doi: 10.3389/fbioe.2023.1227184 (PMC10523579; doi:10.3389/fbioe.2023.1227184)

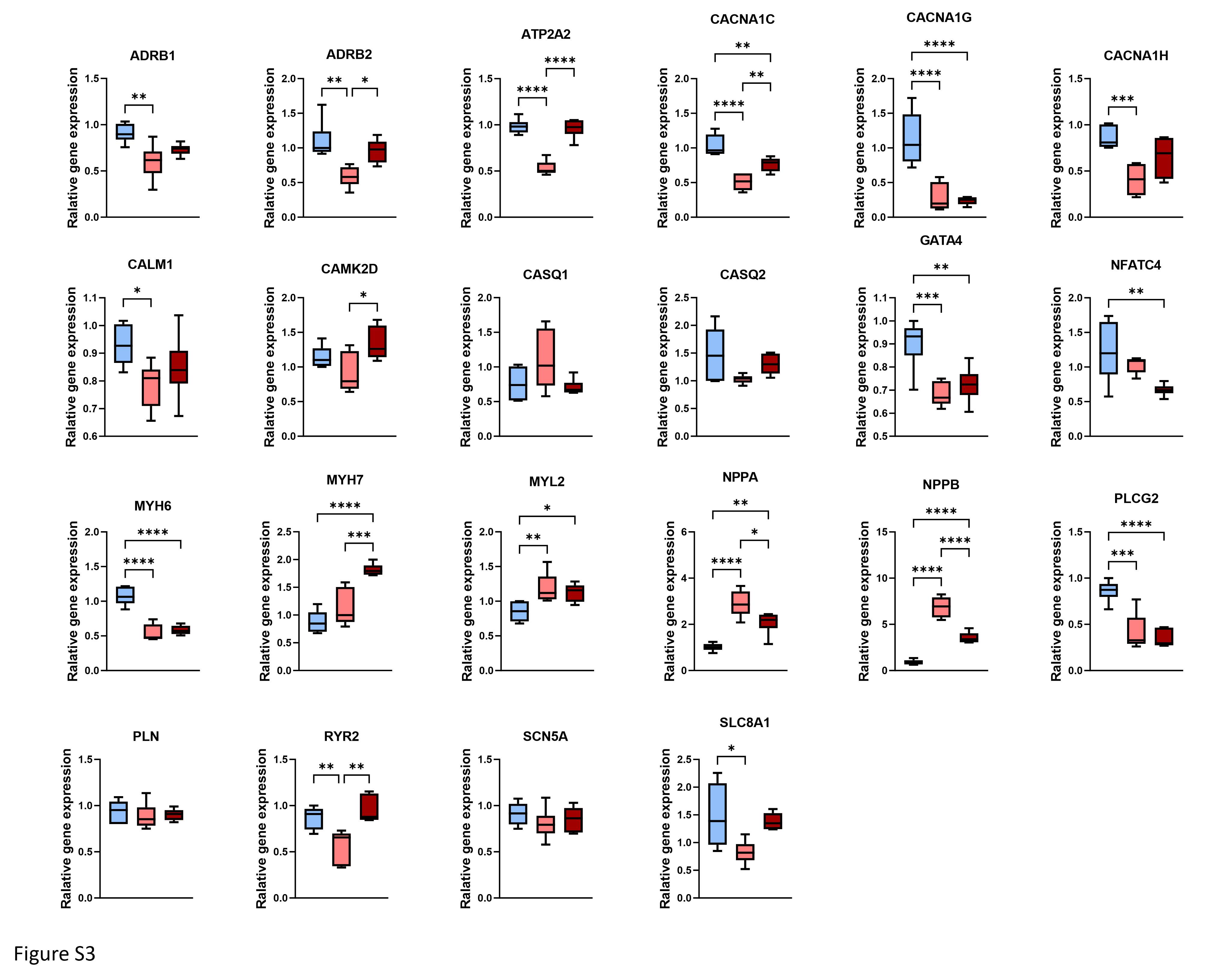

Supplement: Supplementary file 3 [file Image3.TIF]

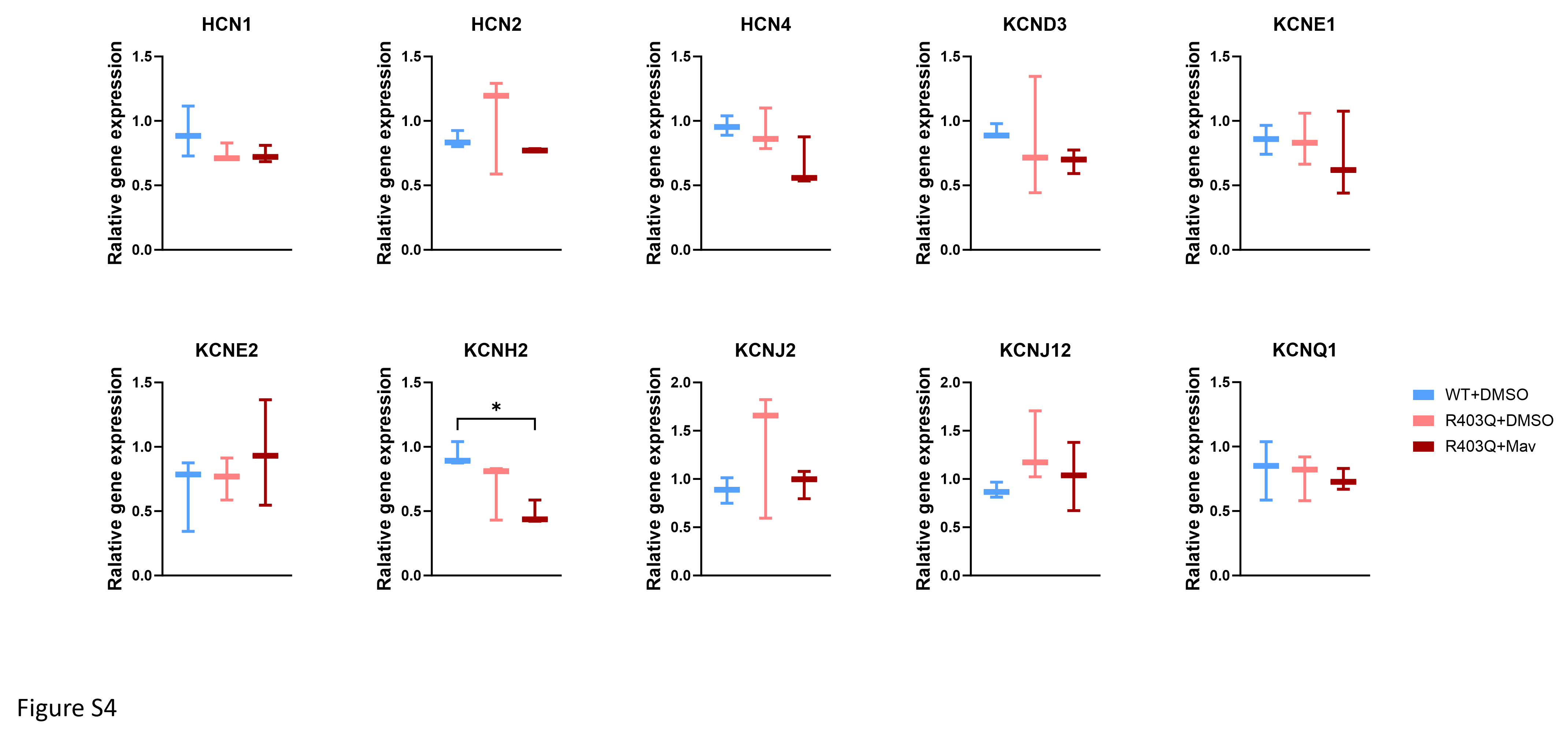

Supplement: Supplementary file 4 [file Image4.TIF]

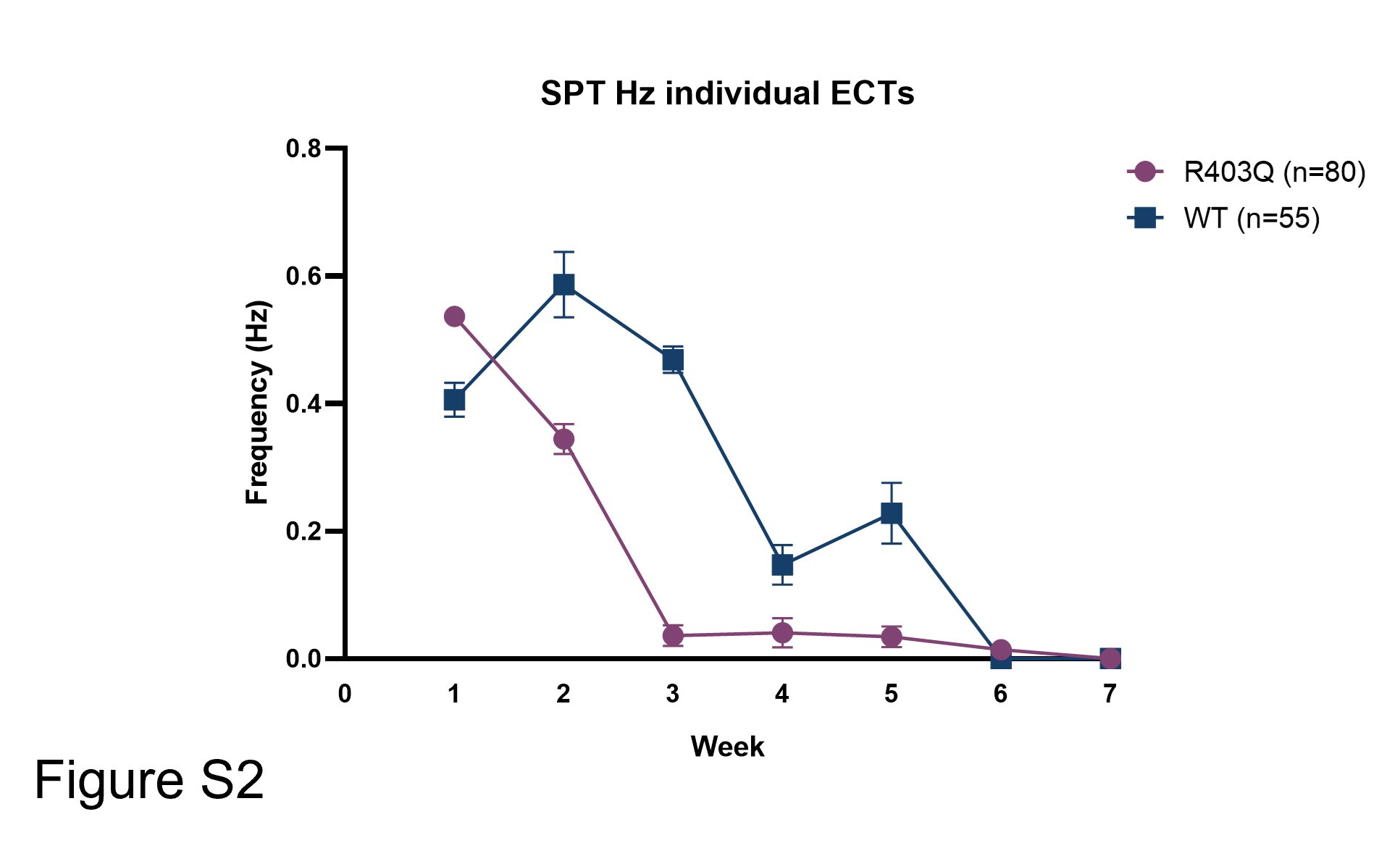

Supplement: Supplementary file 5 [file Image2.TIF]

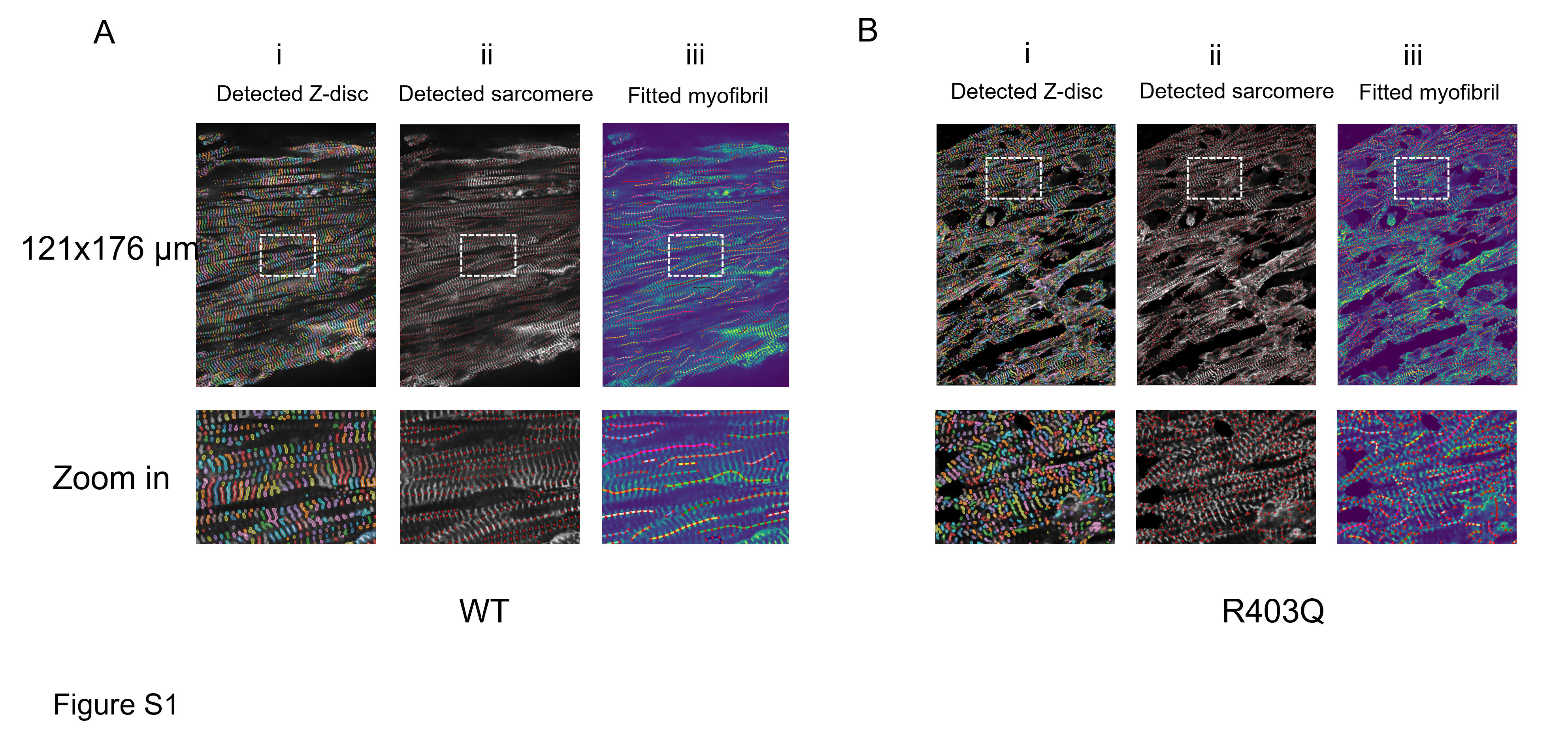

Supplement: Supplementary file 6 [file Image1.TIF]
